# Paraneoplastic Phenomena of Disseminated Intravascular Coagulopathy in Hepatic Angiosarcoma – Rare, Challenging and Fatal. Case Report and Literature Review

**DOI:** 10.15388/Amed.2021.28.2.1

**Published:** 2021-07-29

**Authors:** Sandra Strainienė, Kipras Jauniškis, Ilona Savlan, Justinas Pamedys, Ieva Stundienė, Valentina Liakina, Jonas Valantinas

**Affiliations:** Clinic of Gastroenterology, Nephrourology and Surgery, Centre of Hepatology, Gastroenterology and Dietetics, Institute of Clinical Medicine, Vilnius University, Lithuania https://orcid.org/0000-0003-1884-1353; Vilnius University, Faculty of Medicine, Vilnius, Lithuania https://orcid.org/0000-0002-4318-4431; Clinic of Gastroenterology, Nephrourology and Surgery, Centre of Hepatology, Gastroenterology and Dietetics, Institute of Clinical Medicine, Vilnius University, Lithuania https://orcid.org/0000-0002-3689-5040; National Centre of Pathology, Affiliate of Vilnius University Hospital Santaros Clinics, Vilnius, Lithuania https://orcid.org/000-0001-5263-9891; Clinic of Gastroenterology, Nephrourology and Surgery, Centre of Hepatology, Gastroenterology and Dietetics, Institute of Clinical Medicine, Vilnius University, Vilnius, Lithuania https://orcid.org/0000-0002-2569-3638; Clinic of Gastroenterology, Nephrourology and Surgery, Centre of Hepatology, Gastroenterology and Dietetics, Institute of Clinical Medicine, Vilnius University, Vilnius, Lithuania Department of Chemistry and Bioengineering, Faculty of Fundamental Science, Vilnius Gediminas Technical University, Vilnius, Lithuania https://orcid.org/0000-0001-8685-1292; Clinic of Gastroenterology, Nephrourology and Surgery, Centre of Hepatology, Gastroenterology and Dietetics, Institute of Clinical Medicine, Vilnius University, Vilnius, Lithuania https://orcid.org/0000-0003-4534-2293

**Keywords:** hepatic angiosarcoma, paraneoplastic syndrome, disseminative intravascular coagulopathy, case report, literature review

## Abstract

**Background.:**

Hepatic angiosarcoma is an uncommon, malignant, primary liver tumor, comprising 2% of liver cancers and accounting for < 1% of all sarcomas. Patients usually present with nonspecific symptoms, such as fatigue, weight loss, right upper quadrant pain, anemia, which leads to late diagnosis of an advanced stage tumor. The median life expectancy after the diagnosis of hepatic angiosarcoma is about 6 months, with only 3% of patients surviving more than 2 years. Liver failure and hemoperitoneum are the leading causes of death in patients with liver angiosarcoma. In rarer cases, it might cause paraneoplastic syndromes such as disseminated intravascular coagulopathy. The treatment of angiosarcomas is complicated as there are no established and effective treatment guidelines due to the tumor’s low frequency and aggressive nature.

**Case summary.:**

We present the case of a 68-year old woman who was admitted to the hospital due to fatigue and severe anemia (hemoglobin 65 g/l). Laboratory results also revealed high-grade thrombocytopenia (8 × 10^9^/l). The abdominal ultrasound and computed tomography scan showed multiple lesions throughout the liver, spleen and kidneys. After the histological examination of the liver biopsy, the patient was diagnosed with hepatic angiosarcoma. The treatment with first-line chemotherapy (doxorubicin) was initiated despite ongoing paraneoplastic syndrome – disseminative intravascular coagulopathy. However, the disease was terminal, and the patient died 2 months since diagnosed.

**Conclusions.:**

Hepatic angiosarcoma is a rare and terminal tumor. Therefore, knowledge about its manifestations and effective treatment methods is lacking. Disseminative intravascular coagulopathy is a unique clinical characteristic of angiosarcoma seen in a subset of patients.

## Abstract (lt)

**Paraneoplastinis diseminės intravaskulinės koaguliacijos sindromas sergant kepenų angiosarkoma – reta, sudėtinga ir mirtina patologija. Atvejo aprašymas ir literatūros apžvalga:**

**Santrauka. *****Apžvalga.*** Kepenų angiosarkoma yra retas piktybinis pirminis kepenų navikas, sudaro apie 2 proc. visų kepenų vėžio ir < 1 proc. visų sarkomų atvejų. Liga dažniausiai pasireiškia nespecifiniais simptomais – nuovargiu, svorio kritimu, dešiniojo viršutinio pilvo kvadranto skausmu, anemija. Dėl to kepenų angiosarkoma dažnai diagnozuojama jau pažengusios stadijos. Vidutinė gyvenimo trukmė diagnozavus HA – apie 6 mėnesius ir tik 3 proc. pacientų išgyvena ilgiau nei dvejus metus. Kepenų nepakankamumas ir hemoperitoeumas yra pagrindinės kepenų angiosarkoma sergančių pacientų mirties priežastys. Retesniais atvejais liga gali sukelti paraneoplastinius sindromus, pavyzdžiui, diseminę intravaskulinę koaguliaciją. Kepenų angiosarkomos gydymas yra sudėtingas, nes dėl reto pasireiškimo ir agresyvaus naviko pobūdžio nėra nustatyta bendrų ir efektyvių gydymo gairių.

***Klinikinis atvejis. ***68 metų moteris hospitalizuota dėl progresuojančio nuovargio ir sunkios anemijos (hemoglobinas 65 g/l). Atlikus laboratorinius tyrimus nustatyta didelio laipsnio trombocitopenija (8 × 10^9^/l). Pilvo ultragarsinis tyrimas ir kompiuterinė tomografija parodė daugybinius židininius pakitimus kepenyse, blužnyje ir inkstuose. Atlikus histologinį kepenų biopsijos tyrimą, pacientei diagnozuota kepenų angiosarkoma. Gydymo eigoje išsivystė paraneoplastinis diseminės intravaskulinės koaguliacijos sindromas. Nepaisant to, pradėtas gydymas pirmosios eilės chemoterapija (doksorubicinu). Deja, liga buvo labai sunki ir pacientė mirė praėjus 2 mėnesiams nuo diagnozės nustatymo.

***Išvados. ***Kepenų angiosarkoma yra retas ir mirtinas navikas. Vis dar trūksta žinių apie ligos reiškimąsi ir veiksmingus gydymo metodus. Diseminė intravaskulinė koaguliacija yra unikalus klinikinis angiosarkomos požymis, pasireiškiantis daliai pacientų.

**Raktažodžiai: **kepenų angiosarkoma, paraneoplastinis sindromas, diseminė intravaskulinė koaguliacija, atvejo aprašymas, literatūros apžvalga.

## Introduction

Hepatic angiosarcoma (HA) is a rare, noncirrhotic, primary malignancy of the liver, comprising 2% of liver cancers and accounting for < 1% of all sarcomas [1, 2]. HA predominantly occurs in males, with studies showing a male-to-female ratio of 3 – 4.1 (2:1 in Asian countries) during the 6^th^ and 7^th^ decade of their life [3]. Historically, 25% of HA cases were associated with occupational or medicinal exposure to arsenic, vinyl chloride monomer, thorium dioxide (*Thorotrast*) and radium, but most cases are now considered idiopathic [4, 5].

Patients usually present with vague signs and symptoms of liver disease, often resulting in late diagnosis. The disease might also manifest with acute liver failure or spontaneous rupture of the tumor, but these are rare conditions compared to complaints of nonspecific symptoms, such as right upper quadrant pain, fatigue, weakness and weight loss [1, 5, 6]. Therefore, the tumor is often discovered at an advanced stage with an average diameter of 15 – 65 mm [7].

HA is considered a deep tumor as it is located beneath the superficial fascia. Moreover, it is a rapidly growing and fatal tumor [8]. The median survival after the diagnosis of HA is about 6 months, with only 3% of patients living for more than 2 years [9–11]. Liver failure is the leading cause of death in patients with HA (50% of patients), followed by hemoperitoneum (25% of patients). Other causes include metastatic disease, infection, and, rarely, renal failure and congestive heart failure (3% of deaths) [4, 9–11]. HA most commonly spreads to the lungs, spleen and bones [11–13]. In a subset of patients, the pronounced dysregulation of the coagulation system is detected, also known as disseminated intravascular coagulation (DIC), which is mostly indistinguishable from Kasabach– Merritt syndrome (KMS) [14]. 

The treatment of HA is complicated as there are no established and effective treatment guidelines because of the low frequency and aggressive nature of this particular tumor. Although the most effective treatment is liver resection, it is still challenging since most HA cases are multifocal, involving both lobes of the liver. Moreover, there are no regular effective chemotherapy regimens. There is some promising data in exploring the potential role of the Hippo signaling pathway as a biological treatment of HA, the blockage of the vascular endothelial growth factor (VEGF) pathway, and even the use of beta-blockers [1, 4, 15, 16].

We present the case of hepatic angiosarcoma, which manifested with fatigue and severe anemia, was complicated by DIC syndrome and was fatal despite our best efforts.

## Clinical case

A 68-year-old female was admitted to the centre of hepatology, gastroenterology and dietetics complaining of progressive fatigue for the past 3 weeks. She was consulted by her family doctor several times due to iron deficiency anemia, and 80 mg of ferrous sulfate was prescribed. However, the patient’s condition continued to deteriorate: the weakness progressed, and she reported melena a day before hospitalization. The initial blood test revealed severe anemia with hemoglobin (HgB) of 71 g/L. The patient was referred to the emergency department in suspicion of upper gastrointestinal bleeding. However, esophagogastroduodenoscopy (EGDS) indicated erosive gastropathy with no signs of bleeding. The patient was then admitted to the department of hepatology and gastroenterology for further investigation and treatment.

The patient had a medical history of type 2 insulin-dependent diabetes, hypothyroidism, hypertension, uterine myomatosis and peptic ulcer in the stomach 10 years ago. There was no history of surgical treatment. She denied any harmful habits and claimed to have no allergies to food or medicines. 

On admission, the patient was pale, afebrile (body temperature 36,8º C), with stable hemodynamic function (blood pressure 140/70 mmHg, pulse 90 bpm). Physical examination revealed no signs of cachexia (height 164 cm, weight 68 kg, body mass index 25.28 kg/m^2^) and palpable abdomen pain. Her liver, spleen, and superficial lymph nodes were not enlarged. The patients’ ECOG (*Eastern Cooperative Oncology Group*) performance status was evaluated as 1 point.

The primary laboratory tests performed on admission revealed hemolytic anemia (low hemoglobin, reticulocytosis, elevated lactate dehydrogenase, low haptoglobin), thrombocytopenia, elevated liver enzymes, stage 3A chronic kidney disease, and signs of inflammation. No signs of ferritin, folate, or vitamin B12 deficiency were observed (Table 1).

**Figure 1.**
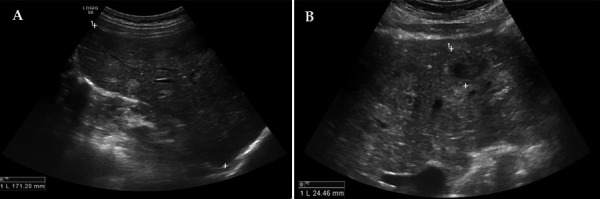
***Abdominal ultrasound images.*** Hepatomegaly and multiple variable echogenicity liver lesions (metastases) throughout all liver segments (the biggest ~25 mm in diameter) (A, B).

The abdominal ultrasound revealed hepatomegaly (right lobe ~171 mm) and multiple variable echogenicity liver metastases (part of which was degrading) throughout all liver segments (the biggest ~25 mm in diameter) (Figure 1, A, B). A nonhomogenous increased uterus with several calcified myomas was also observed. 

**Table 1.** Main laboratory findings

	Value	Normal range
White blood cell count	**10.27**	4.0 – 9.8 x 10^9^/L
Neutrophil count	7.10	2 – 8 x 10^9^/L
Lymphocyte count	2.3	1 – 4 x 10^9^/L
Red blood cell count	**2.0**	3.8 – 5.8 x 10^9^/L
Hemoglobin	**65**	128 – 160 g/L
Hematocrite	**0.195**	0.37-0.47 L/L
Mean cell volume	97.7	80 – 100 fl
Mean cell hemoglobin	**32.6**	27 – 32 pg
Reticulocyte count	**160.07**	30 – 80 x 10^9^/L
Platelet count	**8**	130 – 400 x 10^9^/L
C reactive protein	**12.1**	5 mg/L
Aspartate aminotransferase	**93**	< 40 U/L
Alanine aminotransferase	**139**	< 40 U/L
γ-glutamyl transferase	**403**	≤ 36 U/L
Alkaline phosphatase	**769**	40 – 150 U/L
Albumin	**31.2**	36 – 52 g/L
Lactate dehydrogenase	**629**	125 – 243 U/L
Haptoglobin	**0.34**	0.5 – 2.2 g/L
Total bilirubin	8.4	< 21 µmol
K+	4.6	3.8 –5.3 mmol/L
Na+	139	134 – 145 mmol/L
SPA	115	70 – 130 %
INR by Owren	0.95	0.90 – 1.19
APTT	37.6	30 – 40 s
Creatinine	**107**	62 – 11 µmol/L
Urea	**11.8**	2.5 – 7.5 mmol/L
eGFR (CKD-EPI)	**46**	>90 mL/min/1.73 m^2
Fe	**8.8**	9.5 – 29.9 µmol/L
CA 19.9	4.79	< 37 kU/L
CEA	2.0	< 5 mkg/L
AFP	3.52	0.5 – 5.5 kU/L
Ferritin	254.97	20 – 300 µg/l
Folate	13.48	4.5 – 45.3 nmol/L
Vitamin B12	604	118 – 701 pmol/L

INR, international normalized ratio; SPA, Stago prothrombin assay; APTT, activated partial thromboplastin time; eGFR, estimated glomerular filtration rate; CA 19.9, carbohydrate antigen 19.9; CEA, carcinoembryonic antigen; AFP, alpha-fetoprotein.

**Figure 2.**
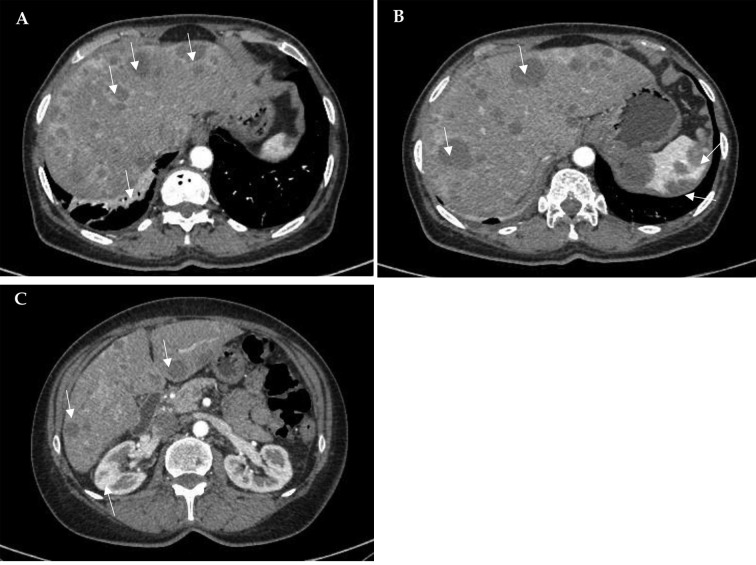
***Computed tomography images.*** Multiple hypodense solid diffusely distributed masses (*arrows*) in the liver (A–C), spleen (B) and kidneys (C); disc atelectasis in the right lung (*arrow*) (A).

Metastatic foci were confirmed by performing a full-body computed tomography (CT) scan, which showed hepatomegaly (right lobe ~184 mm), multiple hypodense solid diffusely distributed masses in the liver (to 3 cm in diameter), spleen (to 3.7 cm in diameter), and kidneys (to 12 mm in diameter), moderate interstitial, para-aortic, paratracheal, and aortocaval lymphadenopathy, and disc atelectasis in the lungs (Figure 2, A–C). 

The primary tumor localization after the CT scan remained unknown. The lesions were differentiated between lymphoma, metastatic disease, granuloma, or infection. Firstly, lymphoma was suspected due to thrombocytopenia, masses in the liver and spleen. The patient was consulted by a hematologist: transfusions of irradiated blood components were intended, and bone marrow biopsy and aspiration were recommended and performed. Trepanobiopsy showed reactive bone marrow changes – bone marrow hyperplasia with massive megacaryopoetic hyperplasia. No metastases were detected. The diagnosis of lymphoma was denied. There were also no visible tumors or other pathological changes found during the colonoscopy. 

**Figure 3.**
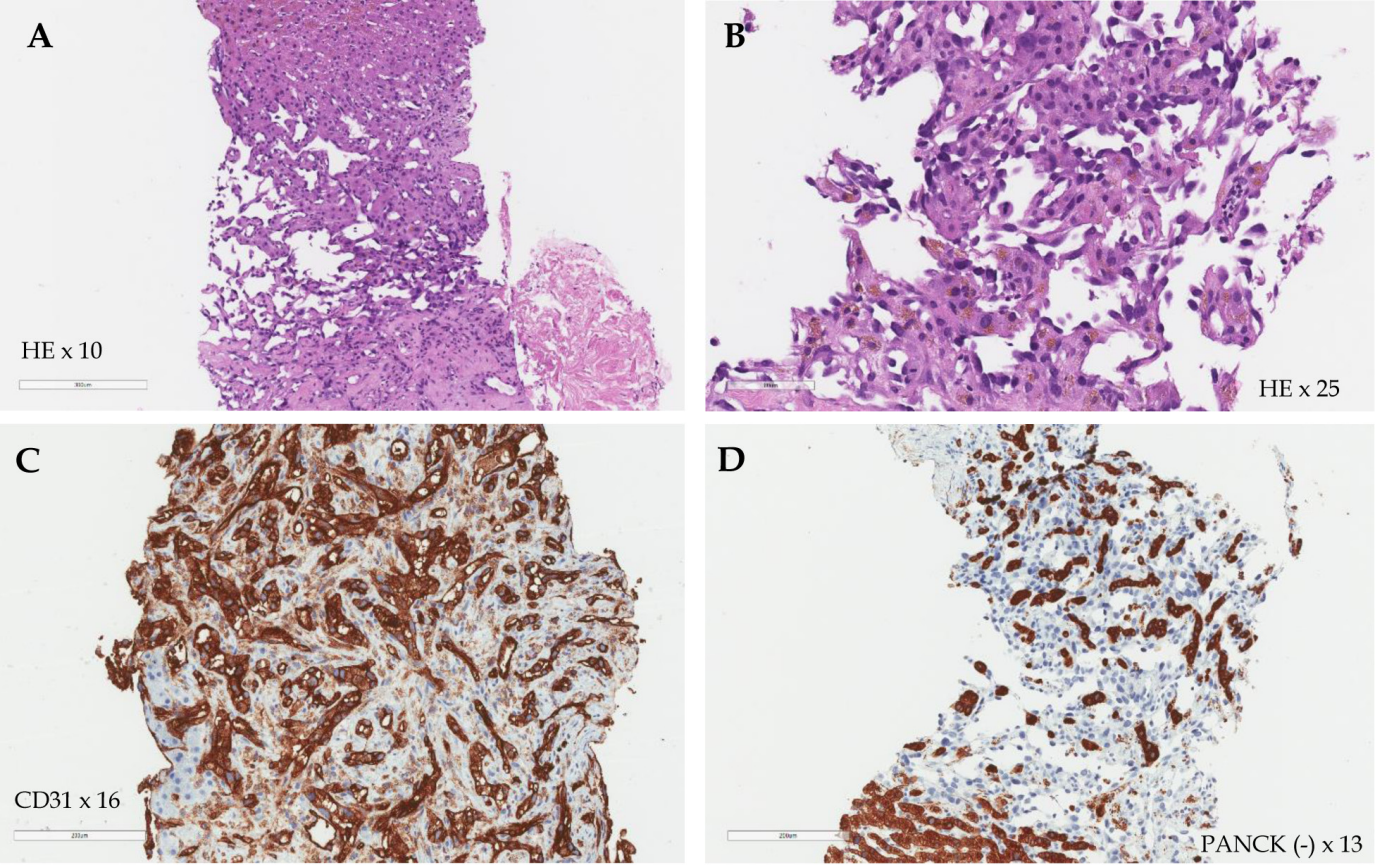
**Histological images of the liver. **The tumor formed by solid, sinusoidal structures of medium caliber cells, featuring eosinophilic cytoplasm and polymorphic, hyperchromatic nuclei (Hematoxylin-eosin (HE) staining, 10 × and 25 × magnitude) (A, B); strong CD31 expression (CD31 staining, 16 × magnitude) (C); negative PANCK reaction (D).

Consequently, a percutaneous focal core needle liver biopsy was performed. The pathological findings confirmed the diagnosis of poorly differentiated (G3) liver angiosarcoma, and the patient was diagnosed with stage IV (cT4N0M1) poorly differentiated (G3) HA with multiple metastases in the spleen and kidneys, severe anemia, and thrombocytopenia. On immunohistochemical examination, the tumor cells showed strong CD31 expression and were negative for CD34, pan Cytokeratin (AE1/AE3) (PANCK) and smooth muscle a-actin (SMA) reactions. Microscopically, the tumor was described as formed by solid, sinusoidal structures of medium caliber cells, featuring eosinophilic cytoplasm (in places with hemosiderin granules) and polymorphic, hyperchromatic nuclei with up to 3 mitoses in the high power field (Figure 3, A–D).

The patient was further referred to the department of oncology and chemotherapy for systemic treatment. By that time, the patient’s platelet count was stabilized from 8 to 344 × 10^9^/L. However, within 4 days, the patient developed multiple hemorrhages on the skin, and her blood tests revealed severe thrombocytopenia – platelet count dropped to 5 × 10^9^/L). Therefore, the chemotherapy was contraindicated. She was treated with platelet transfusions and was also prescribed 20 mg peroral dexamethasone (reduced dose due to insulin-dependent diabetes) for 4 days in suspicion of immune thrombocytopenia. However, this treatment was not effective, and thrombocytopenia (platelet count 6 × 10^9^/L) along with anemia (HgB 73 g/L) remained. Additional laboratory testing revealed hypofi-brinogenemia (fibrinogen 1.22 g/L; normal range 2–4 g/L), normal international normalized ratio, prothrombin time and activated partial thromboplastin ratio levels, high D-dimers (41970 µg/L; normal range < 250 µg/L) and slightly worsened liver function. These changes were compatible with overt DIC. (ISTH criteria 5 points), which might be a paraneoplastic syndrome related to angiosarcoma. 

During the chemotherapy experts’ consultation, it was decided to start systemic first-line treatment with doxorubicin monotherapy urgently, despite remaining thrombocytopenia and anemia (paraneoplastic syndrome). The decision on this particular regimen was made according to the literature review on first-line chemotherapeutic treatment for noncutaneous angiosarcomas. Six cycles of treatment were planned. The patient tolerated the first cycle well, and her platelet level started to rise. However, 11 days after the second treatment cycle with doxorubicin, the patient’s condition worsened tremendously, and she died 2 months after HA diagnosis due to hypovolemic-hemorrhagic shock and DIC despite our best efforts. 

## Discussion

HAs are uncommon liver tumors of endothelial cell origin. It is macroscopically characterized by four growth patterns: multiple nodules, large dominant mass, a mixed pattern of dominant mass with nodules, and, infrequently, a diffusely infiltrating micronodular tumor [4, 12, 17]. Angiosarcomas are infiltrative tumors and usually do not have a capsule or a clear border, separating normal from abnormal tissue [18]. Usually, HA is heterogeneous in appearance with alternating areas of hemorrhagic foci, large intraparenchymal cystic spaces filled with thrombotic content, and gross necrotic areas [2, 19–21].

Microscopically, HA is composed of pleomorphic malignant atypical endothelial cells that can vary in shapes – it can be round, irregular or spindle-shaped [4, 22–24]. The proliferation of neoplastic cells can occur in both single and multiple layers with a possibility to infiltrate along preformed vascular channels, including central veins, portal vein branches and dilated sinusoids [24, 20, 22, 26]. Due to the loss of adjacent hepatocytes, HA cells are prone to form disorganized anastomotic vascular channels, nests, solid nodules or cavernous spaces [1, 4, 16, 17]. This way, HA may mimic cavernous haemangiomas [12]. The surrounding hepatocytes may be hyperplastic or atrophic [7, 24, 27]. The hyperplastic hepatocytes with the irregular dilated lining of the cells and atypical sinusoids are believed to show early changes, while the atrophy of hepatocytes is supposed to indicate a progressive HA [23, 28]. Moreover, in HA cases, areas of hemorrhage, infarction, necrosis and calcifications are also described frequently [21, 25, 29].

Angiosarcomas typically express such endothelial markers as von *Willebrand* factor, CD34, CD31, *Ulex europaeus *agglutinin 1, and VEGF [18]. Other immunohistochemical, tumor and protein markers reported in HA cases include ERG, CD34, FVIII-rA, CD10, CD117, cytokeratin, friend leukemia integration (FLI)-1 and D2-40 [3, 4, 22, 30, 31]. However, these markers are not specific enough since progressive tumor dedifferentiation can lead to a loss of these markers. Therefore they should only be used alongside other investigations to assist diagnosis [4, 18].

In general, sarcomas are not consistently associated with a particular paraneoplastic phenomenon [32]. However, many reports link angiosarcomas to consumptive coagulopathy, also known as KMS, characterized by thrombocytopenia and hyperconsumption of coagulation factors within the tumor. It is thought that platelet trapping and physiological derangements of malignant endothelial cells promote platelet adhesion and activation [14, 33]. Moreover, as in our case, angiosarcomas may even progress to DIC, potentially by induction of tissue factor and thromboplastin released by malignant epithelial cells, the release of clotting factors as a result of decreased blood supply flow triggering blood cell trapping and lysis, and exposure of the endothelial basement membrane cells [24, 20, 33]. A report by Farid et al. provided data that 17% of angiosarcoma patients in their series developed DIC (n=42), with all patients’ data meeting the ISTH criteria for overt DIC [32].

Diagnostic assessment of HA includes liver biopsy for histological confirmation and magnetic resonance imaging or CT to precisely portray the primary lesion’s extent [18]. PET imaging may be useful to detect metastases before planned radical surgical treatment[18, 34]. Angiosarcomas can spread through the lymphatic system. However, the value of sentinel-lymph-node biopsy is unknown. The primary lesion size and the presence or absence of distal metastases are the handiest factors that determine available treatment options [18].

Clinical staging of angiosarcoma is based on the American Joint Committee on Cancer Staging System for soft tissue sarcoma, consisting of tumor size, depth, presence or absence of regional lymph nodes, and distant metastases. Since angiosarcomas are high-grade tumors by definition, histological grading is not used in its staging [18].

To this date, the most favorable treatment of HA is radical surgical resection of the tumor with resection margin being R0 – no cancer cells seen microscopically at the primary tumor site [4, 9, 31]. A combination of adjuvant chemotherapy with surgical treatment gives the highest chance of cure, with the reported median survival of approximately 17 months [4, 9]. Due to the high HA recurrence rates, liver transplantation is contraindicated. The median post-transplant survival is less than 7 months [4]. Orlando et al. stated that no patient has survived more than 23 months after receiving a liver transplant [35]. Since HA has a feature of intrinsic radioresistance, radiotherapy has been abandoned as a potential treatment option [4, 13]. 

Transarterial embolization (TAE) and transarterial chemoembolization (TACE) in HA emergency use and palliative care are also highly discussed treatment options. TAE is an effective technique for stopping deadly intra-abdominal bleeding due to the rupture of the tumor [4, 9, 36]. TACE is mainly used as palliative therapy for patients with unresectable tumors [36]. Park et al. described a few TACE cases where emulsion of iodized oil and cisplatin helped the patients survive up to one year [37]. 

Many chemotherapy regimens have been described in various articles, but no routine has proven to be notably superior to the other [4]. There is no consensus regarding chemotherapeutic treatment of HA due to the limited amount of clinical trials with HA patients [10]. The literature review on first-line chemotherapeutic treatment for non-cutaneous angiosarcomas suggests taxanes, doxorubicin, liposome doxorubicin and ifosfamide as a primary choice of chemotherapy agents [38]. Cao et al. state that anthracycline and taxane chemotherapy are considered active and recommended treatment choices [16]. However, Alliot et al. believe that single-agent chemotherapy regimens are not effective enough and show disappointing results [25]. Kim et al. have demonstrated improved survival in 50% (n=2) of patients treated with the combination of 5-fluorouracil, carboplatin, doxorubicin and ifosfamide [13]. Other authors displayed a partial response with mesna, doxorubicin, ifosfamide and dacarbazine regimens [39]. However, due to elderly age and overlapping toxicity of treatment combinations, the patients are usually given lower doses to avoid underlying complications [16]. Therefore, given the nature of HA, which includes late diagnosis and natural heterogeneity of the tumor, both – single agent and combined chemotherapy – do not significantly alter the course of this aggressive tumor. However, it is still considered the main treatment option for metastatic HA [16, 40].

Hopefully, the potential role of the Hippo signaling pathway, responsible for the regulation of cell proliferation and apoptosis, was explored in the biological treatment of angiosarcomas showing some promising results [4, 15]. Yes-associated protein (YAP), an oncogene involved in the mentioned pathway, and CD31 regulate endothelial cell function and redox status via YAP [38]. Angiosarcoma cells were subclassified based on the phenotypical expression, and the results showed that CD31low was more common in angiosarcoma cases than CD31high, and it was associated with YAP increase, making the tumor more chemoresistant to different agents, for example, doxorubicin [4, 15]. Venkataramani et al. demonstrated *in vitro *that pazopanib, an effective YAP inhibitor, was effective when used with doxorubicin and resulted in resensitization of CD31low to chemotherapy [15]. Liau et al. also described the mechanism of alternative lengthening of telomeres (ALT) that was highly associated with HA, since 30% of primary angiosarcomas were ALT positive. With two-thirds of the population being positive for ALT, they reported that ALT-positive cells were sensitive to ataxia telangiectasia and Rad3 related (ATR) kinase inhibitors [39]. However, further *in vivo *trials are required to outline the benefits and efficacies of ATR kinase inhibitor and YAP inhibitor therapy combined with chemotherapy to treat HA [4,15,39].

Moreover, other authors discuss the potential of targeted therapy and beta-blockers in the treatment of HA [16]. The overexpression of VEGF is considered the most significant angiogenic factor in different subtypes of sarcomas, including angiosarcomas [16, 41, 42]. Therefore, a VEGF and VEGF receptor signaling pathway inhibitor, that is tyrosine kinase inhibitor has been implemented as targeted therapy [16]. Sorafenib and pazopanib were used in phase II clinical trials by Penel et al., and their suppressive activity was confirmed to be useful in the treatment of angiosarcoma, despite disappointing responses in some other presented cases [43–45]. All things considered, the VEGF pathway blockage is still a promising treatment option that needs further research [16].

Furthermore, Rains et al. presented retrospective studies with large patient cohorts that provide data about the expression of high levels of beta-adrenergic receptors in malignant vascular tumors like HA [16, 46]. Amaya et al. reported that nonselective beta-blockers, such as propranolol, can improve the outcomes of the patients with metastatic angiosarcoma, extending the median progression-free survival to 9 months and median overall survival reaching 36 months [47]. Besides, a case series of 7 patients with angiosarcoma, who received a combination between bi-daily propranolol (40 mg) and weekly metronomic vinblastine (6 mg/m^2^), reported a 100% response rate with a median progression-free period of 11 months [48].

With the advances in genetic testing, whole exome sequencing (WES) of fresh tumor specimens via a bioinformatics pipeline may help identify potential actionable chemotherapy agents for angiosarcomas and may lead to developing personalized cancer therapy in the future. A pilot, prospective, nonrandomized control experimental study by Calvert et al. analyzed 12 patients with metastatic bone or soft tissue sarcoma who had failed first-line chemotherapy treatment [49]. One part of the surgical tumor biopsy material was used for WES, and the remaining part was implanted subcutaneously in immunodeficient mice. Patient-derived xenograft (PDX) models exhibiting successful growth underwent WES as well. The results of WES were analyzed using a bioinformatics pipeline to identify mutations conferring susceptibility to common chemotherapeutic agents. As a result, WES identified potential actionable therapeutics in all 12 patients with successfully established PDX tumor models in 7 patients. Significant variation in predicted therapeutics was demonstrated between three PDX samples and their matched tumor samples [49]. This trial is an example of a bright glimpse into modern personalized cancer management as genetic testing gets more available every year [50].

We summarized the available data from the published HA case reports focusing on treatment method and survival in Table 2.

**Table 2.** Cases series of HA described in different literature

Author (year of publication)	Number of cases	Treatment method	Median survival	Additional comments
This case	1	Chemotherapy using doxorubicin	2 months	DIC
Zhu et al., 2015 [2]	2	Surgery	12 months	–
Bruegel et al., 2013 [17]	7	1 surgery 4 chemotherapy*	16 months	*No information available, as patient started or continued treatment in another hospital
Duan et al., 2012 [51]	6	5 surgeries, 1 surgery + chemotherapy	40.5 months	–
Huang et al., 2011 [3]	9	3 surgeries + chemotherapy, 1 operation + TAE, 1 TAE + TACE, 4 conservative	4 months	–
Chi et al., 2011 [52]	7	3 surgeries, 2 liver transplantations, 2 conservative	6.5 months	–
Zhou et al., 2010 [53]	6	1 operation, 5 surgeries + TACE	15.5 months	–
Matthaei et al., 2009 [54]	5	Surgeries	30 months	–
Kim et al., 2009 [13]	5	4 chemotherapy, 1 conservative	3 months	–
Kim et al., 2009 [13]	2	Chemotherapy with docetaxel, cisplatin, 5-fluorouracil	2 months	–
Kim et al., 2009 [13]	2	1 combined chemotherapy with docetaxel, cisplatin, 5-fluorouracil, ifosfamide and doxorubicin. 1 combined chemotherapy with ifosfamide, doxorubicin, paclitaxel and bevacizumab.	11.6 months	–
Egea et al., 2009 [55]	2	2 conservative	< 7 days*	*1 patient died of liver rupture
Park et al., 2009 [37]	6	4 TACE, 2 TAE	3.5 months	–
Fury et al., 2005 [56]*	41	Chemotherapy with paclitaxel	Median progression-free survival – 4 months	*Case reports describing angiosarcoma treatment – not specifically HA. Significant difference in taxane response for disease above and below the neck (6.8 vs 2.8 months)
Fury et al., 2005 [56]*	30	Chemotherapy with doxorubicin	Median progression-free survival – 3.7-5.4 months	*Case reports describing angiosarcoma treatment – not specifically HA
Ozden et al., 2003 [57]	1	Surgery + TACE	64 months	–
Vennarecci et al., 1997 [58]	2	Liver transplantation*	4 months	*Recurrence – 100 %
Tordjman et al., 1995 [59]	1	TACE	15 months	–
Penn I., 1991 [60]	6	Liver transplantation*	5.7 months	*Recurrence – 100 %

TACE, transarterial chemoembolization; TAE, transarterial embolization

In our case, the patient was previously conditionally healthy and did not address any disturbing symptoms until 3 weeks to admission. She experienced some nonspecific symptoms, such as fatigue and anemia, which were first related to iron deficiency. As the patient’s condition deteriorated and she had an episode of melena, she was admitted to the emergency department, where severe anemia and thrombocytopenia were observed. Firstly, bleeding from the upper gastrointestinal tract was suspected. However, further examination revealed no signs of bleeding in EGDS and multiple lesions in the liver, kidneys and spleen. It was differentiated between lymphoma, metastatic disease, granuloma, or infection. Firstly, lymphoma was suspected due to thrombocytopenia and masses in the liver and spleen. However, liver biopsy indicated stage IV liver angiosarcoma. We tried to manage the patient’s thrombocytopenia with numerous platelet transfusions and dexamethasone (in suspicion of immune thrombocytopenia) unsuccessfully. Later on, additional testing showed signs of overt DIC, which was considered a paraneoplastic syndrome related to angiosarcoma. Despite this condition, it was decided to initiate urgent first-line treatment with doxorubicin monotherapy. However, she only survived 2 cycles of chemotherapy and died due to hypovolemic-hemorrhagic shock and DIC despite our best efforts. Hepatic angiosarcoma is a very challenging condition. It has no specific symptoms and is usually diagnosed in advanced stages leading to poor prognosis and severe complications. In our case, the disease was complicated with the paraneoplastic syndrome of DIC, which worsened the overall patient’s prognosis and possible treatment options.

## Conclusions

HA is a rare, primary malignancy of the liver with an extremely poor prognosis. Patients usually present with nonspecific symptoms. In rarer cases, paraneoplastic syndromes, such as DIC, may occur and exaggerate poor outcomes. No effective treatment has been recommended to date. However, there are some promising results showing the potential role of the Hippo signaling pathway and the effectiveness of the biological treatment.

## References

[ref1] Chaudhary P, Bhadana U, Singh RAK, Ahuja A. Primary hepatic angiosarcoma. Eur J Surg Oncol. 2015;41(9):1137– 1343. doi: 10.1016/j.ejso.2015.04.022. 26008857

[ref2] Zhu YP, Chen YM, Matro E, Chen RB, Jiang ZN, Mou YP, et al. Primary hepatic angiosarcoma: A report of two cases and literature review. World J Gastroenterol. 2015;21(19):6088–6096. doi: 10.3748/wjg.v21.i19.6088. PMID:. 26019478 PMC4438048

[ref3] Huang NC, Wann SR, Chang HT, Lin SL, Wang JS, Guo HR. Arsenic, vinyl chloride, viral hepatitis, and hepatic angiosarcoma: A hospital-based study and review of literature in Taiwan. BMC Gastroenterol. 2011;11:142. doi: 10.1186/1471-230X-11-142. PMID:. 22200164 PMC3280174

[ref4] Chen N, SHeng J, Jung J. Primary hepatic angiosarcoma : a brief review of the literature. Eur Med J. 2018;1(6):64–71.

[ref5] Huang N-C, Kuo Y-C, Chiang J-C, Hung S-Y, Wang H-M, Hung Y-M, et al. Hepatic angiosarcoma may have fair survival nowadays. Medicine. 2015; 94(19):e816. doi: 10.1097/MD.0000000000000816. 25984668 PMC4602568

[ref6] Molina E, Hernandez A. Clinical manifestations of primary hepatic angiosarcoma. Dig Dis Sci. 2003;48(4):677–682. 10.1023/A:1022868221670. 12741455

[ref7] Huerta-Orozco LD, Leonher-Ruezga KL, Ramírez-González LR, Hermosillo-Sandoval JM, Sandoval-Alvarado JDJ, Morán-Galaviz RE. Hepatic angiosarcoma and liver transplantation: case report and literature review. Cirugia y Cirujanos. 2015;83(6):510–515. doi: 10.1016/j.circir.2015.05.027. 26144270

[ref8] Huang IH, Wu YIY, Huang TZUC, Chang WEIKUO, Chen JIAH. Statistics and outlook of primary hepatic angiosarcoma based on clinical stage. 2016;(325):3218–3222. doi: 10.3892/ol.2016.4348. PMC4840876 27123094

[ref9] Zheng YW, Zhang XW, Zhang JL, Hui ZZ, Du WJ, Li RM, Ren XB. Primary hepatic angiosarcoma and potential treatment options. J Gastroenterol Hepatol. 2014;29(5):906–911. doi: 10.1111/jgh.12506. 24372769

[ref10] Timaran CH, Grandas OH, Bell JL. Hepatic angiosarcoma: long-term survival after complete surgical removal. Am Surg. 2000;66(12):1153–1157. PMID:. 11149588

[ref11] Locker GY, Doroshow JH, Zweilling LA, Chabner BA. The Clinical Features of Hepatic Angiosarcoma: A Report of four cases and a review of the english literature. Medicine. 1979;58(1):48–64. doi: 10.1097/00005792-197901000-00003. 368508

[ref12] Koyama T, Fletcher JG, Johnson CD, Kuo MS, Notohara K, Burgart LJ. Primary hepatic angiosarcoma: Findings at CT and MR imaging. Radiology. 2002;222(3):667–673. doi: 10.1148/radiol.2223010877. 11867783

[ref13] Kim HR, Rha SY, Cheon SH, Roh JK, Park YN, Yoo NC. Clinical features and treatment outcomes of advanced stage primary hepatic angiosarcoma. Ann Oncol. 2009;20(4):780–787. doi: 10.1093/annonc/mdn702. PMID:. 19179547

[ref14] Wadhwa S, Kim TH, Lin L, Kanel G, Saito T. Hepatic angiosarcoma with clinical and histological features of Kasabach-Merritt syndrome. World J Gastroenterol. 2017;23(13):2443. doi: 10.3748/wjg.v23.i13.2443. PMID:. 28428724 PMC5385411

[ref15] Venkataramani V, Kuffer S, Cheung KCP, Jiang X, Trumper L, Wulf GG, et al. CD31 expression determines redox status and chemoresistance in human angiosarcomas. Clin Cancer Res. 2018;24(2):460–473. doi: 10.1158/1078-0432.CCR-17-1778. PMID:. 29084920 PMC7464789

[ref16] Cao J, Wang J, He C, Fang M. Angiosarcoma: a review of diagnosis and current treatment. Am J Cancer Res. 2019;9(11):2303–2313. PMID:. 31815036 PMC6895451

[ref17] Bruegel M, Muenzel D, Waldt S, Specht K, Rummeny EJ. Hepatic angiosarcoma: Cross-sectional imaging findings in seven patients with emphasis on dynamic contrast-enhanced and diffusion-weighted MRI. Abdom Imaging. 2013;38(4):745–754. doi: 10.1007/s00261-012-9967-2. 23223833

[ref18] Young RJ, Brown NJ, Reed MW, Hughes D, Woll PJ. Angiosarcoma. Lancet Oncol. 2010;11(10):983–991. doi: 10.1016/S1470-2045(10)70023-1. 20537949

[ref19] Bioulac-Sage P, Laumonier H, Laurent C, Blanc JF, Balabaud C. Benign and malignant vascular tumors of the liver in adults. Semin Liver Dis. 2008;28(3):302–314. doi: 10.1055/s-0028-1085098. PMID:. 18814083

[ref20] Mazharuddin S, Podduturi V, Guileyardo JM, Cooper B. Hepatic angiosarcoma associated with disseminated intravascular coagulation. Baylor Univ Med Cent Proc. 2015;28(1):54–56. 10.1080/08998280.2015.11929186. PMC4264711 25552799

[ref21] Ling W, Qiu T, Ma L, Lei C, Luo Y. Contrast-enhanced ultrasound in diagnosis of primary hepatic angiosarcoma. J Med Ultrason. 2017;44(3):267–70. doi: 10.1007/s10396-016-0761-6. 27909829

[ref22] Wang ZB, Yuan J, Chen W, Wei LX. Transcription factor ERG is a specific and sensitive diagnostic marker for hepatic angiosarcoma. World J Gastroenterol. 2014;20(13):3672–3679. doi: 10.3748/wjg.v20.i13.3672. PMID:. 24707153 PMC3974537

[ref23] Ito Y, Kojiro M, Nakashima T, Mori T. Pathomorphologic characteristics of 102 cases of thorotrast-related hepatocellular carcinoma, cholangiocarcinoma, and hepatic angiosarcoma. Cancer. 1988;62(6):1153–1162. doi: 10.1002/1097-0142(19880915)62:6>1153::AID-CNCR2820620619>3.0.CO;2-I. PMID:. 2457426

[ref24] Whelan JG, Creech JL, Tamburro CH. Angiographic and radionuclide characteristics of hepatic angiosarcoma found in vinyl chloride workers. Radiology. 1976;118(3):549–557. doi: 10.1148/118.3.549. 943124

[ref25] Alliot C, Tribout B, Barrios M, Gontier MF. Angiosarcoma variant of Kasabach-Merritt syndrome. Eur J Gastroenterol Hepatol. 2001;13(6):731–734. doi: 10.1097/00042737-200106000-00020. PMID:. 11434603

[ref26] Lee SW, Song CY, Gi YH, Kang SB, Kim YS, Nam SW, et al. Hepatic angiosarcoma manifested as recurrent hemoperitoneum. World J Gastroenterol. 2008;14(18):2935–2938. 10.3748/wjg.14.2935. PMID:. 18473427 PMC2710744

[ref27] Thapar S, Rastogi A, Ahuja A, Sarin S. Angiosarcoma of the liver: Imaging of a rare salient entity. J Radiol Case Rep. 2014;8(8):24–32. doi: 10.3941/jrcr.v8i8.1693. PMID:. 25426242 PMC4242144

[ref28] Berk PD. Vinyl Chloride-Associated Liver Disease. Ann Intern Med. 1976;84(6):717. doi:10.7326/0003-4819-84-6-717. 945708 10.7326/0003-4819-84-6-717

[ref29] Cawich SO, Thomas D, Ragoonanan V, Naraynsingh V. The hanging manoeuver to complete liver resection for a locally advanced angiosarcoma: A case report. Int J Surg Case Rep. 2015;16:52–55. doi: 10.1016/j.ijscr.2015.09.006. 26413923 PMC4643337

[ref30] Akdoğan M, Gürakar A, Sharago S, El-Sahwi K, Carlson J, Sebastian A, et al. Unusual presentation of hepatic vascular tumors as fulminant hepatic failure. Turk J Gastroenterol. 2002;13(4):216–220. PMID:. 16378309

[ref31] Prenen H, Smeets D, Mazzone M, Lambrechts D, Sagaert X, Sciot R, et al. Phospholipase C gamma 1 (PLCG1) R707Q mutation is counterselected under targeted therapy in a patient with hepatic angiosarcoma. Oncotarget. 2015;6(34):36418–36425. doi: 10.18632/oncotarget.5503. PMID:. 26474454 PMC4742186

[ref32] Farid M, Ahn L, Brohl A, Cioffi A, Maki RG. Consumptive coagulopathy in angiosarcoma: A recurrent phenomenon? Sarcoma. 2014;2014. doi: 10.1155/2014/617102. PMC3945465 24693222

[ref33] Fields BKK, Matcuk GR, Lai D, Lee A, Dwabe S, Hanlon C, et al. Primary hepatic angiosarcoma: A casebased discussion of unique presentations and extrahepatic manifestations. Curr Probl Cancer Case Reports. 2020;1(5):100012. doi: 10.1016/j.cpccr.2020.100012.

[ref34] Woll PJ, Reichardt P, Le Cesne A, Bonvalot S, Azzarelli A, Hoekstra HJ, et al. Adjuvant chemotherapy with doxorubicin, ifosfamide, and lenograstim for resected soft-tissue sarcoma (EORTC 62931): A multicentre randomised controlled trial. Lancet Oncol. 2012;13(10):1045–1054. doi: 10.1016/S1470-2045(12)70346-7. PMID:. 22954508

[ref35] Orlando G, Adam R, Mirza D, Soderdahl G, Porte RJ, Paul A, et al. Hepatic hemangiosarcoma: An absolute contraindication to liver transplantation the european liver transplant registry experience. Transplantation. 2013;95(6):872–877. doi: 10.1097/TP.0b013e318281b902. PMID:. 23354302

[ref36] Tripke V, Heinrich S, Huber T, Mittler J, Hoppe-Lotichius M, Straub BK, et al. Surgical therapy of primary hepatic angiosarcoma. BMC Surg. 2019;19(1):1–6. doi: 10.1186/s12893-018-0465-5. PMID:. 30630447 PMC6329081

[ref37] Park YS, Kim JH, Kim KW, Lee IS, Yoon HK, Ko GY, et al. Primary hepatic angiosarcoma: imaging findings and palliative treatment with transcatheter arterial chemoembolization or embolization. Clin Radiol. 2009;64(8):779–785. doi: 10.1016/j.crad.2009.02.019. PMID:. 19589416

[ref38] Oku Y, Nishiya N, Shito T, Yamamoto R, Yamamoto Y, Oyama C, et al. Small molecules inhibiting the nuclear localization of YAP/TAZ for chemotherapeutics and chemosensitizers against breast cancers. FEBS Open Bio. 2015;5:542–549. doi: 10.1016/j.fob.2015.06.007. PMC4506957 26199863

[ref39] Liau J-Y, Tsai J-H, Yang C-Y, Lee J-C, Liang C-W, Hsu H-H, et al. Alternative lengthening of telomeres phenotype in malignant vascular tumors is highly associated with loss of ATRX expression and is frequently observed in hepatic angiosarcomas. Hum Pathol. 2015;46(9):1360–6. doi: 10.1016/j.humpath.2015.05.019. PMID:. 26190196

[ref40] Averbukh LD, Mavilia MG, Einstein MM. Hepatic Angiosarcoma: A Challenging Diagnosis. Cureus. 2018;10(9): e3283. doi: 10.7759/cureus.3283. 30443453 PMC6235643

[ref41] Kieran MW, Kalluri R, Cho Y-J. The VEGF Pathway in cancer and disease: responses, Resistance, and the Path Forward. Cold Spring Harb Perspect Med. 2012;2(12):a006593–a006593. doi: 10.1101/cshperspect.a006593. PMID:. 23209176 PMC3543071

[ref42] Cuppens T, Tuyaerts S, Amant F. Potential Therapeutic Targets in Uterine Sarcomas. Sarcoma. 2015;2015. doi: 10.1155/2015/243298. PMC4632006 26576131

[ref43] Penel N, Ray-Coquard I, Bal-Mahieu C, Chevreau C, Le Cesne A, Italiano A, et al. Low level of baseline circulating VEGF-A is associated with better outcome in patients with vascular sarcomas receiving sorafenib: an ancillary study from a phase II trial. Target Oncol. 2014;9(3):273–277. doi: 10.1007/s11523-013-0299-0. PMID:. 24218035

[ref44] Hoang NT, Acevedo LA, Mann MJ, Tolani B. A review of soft-tissue sarcomas: Translation of biological advances into treatment measures. Cancer Manag Res. 2018;10:1089–1114. 10.2147/CMAR.S159641. 29785138 PMC5955018

[ref45] Ravi V, Sanford EM, Wang WL, Ross JS, Ramesh N, Futreal A, et al. Antitumor response of VEGFR2- and VEGFR3-amplified angiosarcoma to pazopanib. J Natl Compr Cancer Netw. 2016;14(5):499–502. doi: 10.6004/jnccn.2016.0058. PMID:. 27160228

[ref46] Rains SL, Amaya CN, Bryan BA. Beta-adrenergic receptors are expressed across diverse cancers. Oncoscience. 2017;4(7–8):95–105. doi: 10.18632/oncoscience.357. 28966942 PMC5616202

[ref47] Amaya CN, Perkins M, Belmont A, Herrera C, Nasrazadani A, Vargas A, et al. Non-selective beta blockers inhibit angiosarcoma cell viability and increase progression free- and overall-survival in patients diagnosed with metastatic angiosarcoma. Oncoscience. 2018;5(3–4):109–119. doi: 10.18632/oncoscience.413. 29854879 PMC5978448

[ref48] Pasquier E, André N, Street J, Chougule A, Rekhi B, Ghosh J, et al. Effective management of advanced angiosarcoma by the synergistic combination of propranolol and vinblastine-based metronomic chemotherapy: a bench to bedside study. EBioMedicine. 2016;6:87–95. doi: 10.1016/j.ebiom.2016.02.026. PMID:. 27211551 PMC4856748

[ref49] Calvert N, Wu J, Sneddon S, Woodhouse J, Carey-Smith R, Wood D, et al. The use of whole exome sequencing and murine patient derived xenografts as a method of chemosensitivity testing in sarcoma. Clin Sarcoma Res. 2018;8(1):4. 10.1186/s13569-018-0090-1. 29541442 PMC5842605

[ref50] Schwarze K, Buchanan J, Taylor JC, Wordsworth S. Are whole-exome and whole-genome sequencing approaches cost-effective? A systematic review of the literature. Genet Med. 2018;20(10):1122–1130. doi: 10.1038/gim.2017.247. PMID:. 29446766

[ref51] Duan XF, Li Q. Primary hepatic angiosarcoma: A retrospective analysis of 6 cases. J Dig Dis. 2012;13(7):381–385. doi: 10.1111/j.1751-2980.2012.00600.x. PMID:. 22713088

[ref52] Chi T, Yang Z, Xue H, Lü K, Feng R, Xu H, et al. Diagnosis and treatment of primary hepatic angiosarcoma: a report of 7 cases with a literature review. Zhonghua Yi Xue Za Zhi. 2011;91(24):1694–7. doi: 10.3760/cma.j.issn.0376-2491.2011.24.010. PMID:. 21914319

[ref53] Zhou Y-M, Li B, Yin Z-M, Xu F, Wang B, Xu W, et al. Results of hepatic resection for primary hepatic angiosarcoma in adults. Med Sci Monit. 2010 ;16(2):CR61–66. PMID:. 20110916

[ref54] Matthaei H, Krieg A, Schmelzle M, Boelke E, Poremba C, Rogiers X, et al. Long-term survival after surgery for primary hepatic sarcoma in adults. Arch Surg. 2009;144(4):339–344. doi: 10.1001/archsurg.2009.30. PMID:. 19380647

[ref55] Egea Valenzuela J, López Poveda MJ, Pérez Fuenzalida FJ, Garre Sánchez C, Martínez Barba E, Carballo Alvarez F. Hepatic angiosarcoma. Presentation of two cases. Rev Esp Enferm Dig. 2009;101(6):430–437. doi: 10.4321/s1130-01082009000600010. PMID:. 19630468

[ref56] Fury MG, Antonescu CR, Van Zee K, Brennan ME, Maki RG. A 14-year retrospective review of angiosarcoma: clinical characteristics, prognostic factors, and treatment outcomes with surgery and chemotherapy. Cancer J. 2005;11(3):241–247. doi: 10.1097/00130404-200505000-00011. PMID:. 16053668

[ref57] Ozden I, Bilge O, Erkan M, Cevikbaş U, Acarli K. Five years and 4 months of recurrence-free survival in hepatic angiosarcoma. J Hepatobiliary Pancreat Surg. 2003;10(3):250–252. doi: 10.1007/s00534-003-0849-4. PMID:. 14605984

[ref58] Vennarecci G, Ismail T, Gunson B, McMaster P. Primary angiosarcoma of the liver. Minerva Chir. 1997;52(10):1141–1146. PMID:. 9471563

[ref59] Tordjman R, Eugène C, Clouet O, Wesenfelder L, Collet C, Bergue A. Hepatosplenic angiosarcoma complicated by hemoperitoneum and disseminated intravascular coagulation. Treatment by arterial embolization and chemotherapy. Gastroenterol Clin Biol. 19(6–7):625–628. PMID:. 7590030

[ref60] Penn I. Hepatic transplantation for primary and metastatic cancers of the liver. Surgery. 1991;110(4):726–735. PMID:. 1656538

